# A clinical prediction tool for ocular hypertension following silicone oil tamponade in rhegmatogenous retinal detachment

**DOI:** 10.1186/s12886-026-04728-6

**Published:** 2026-03-14

**Authors:** Huan Ju, Xuefeng Qin, Xing Wang, Lingyi Jin, Yuanyuan Wu, Qingyue Luo, Huan Zhou, Ni Zhang, Hui Peng

**Affiliations:** 1 Department of Ophthalmology, The First Affiliated Hospital of Chongqing Medical University, Chongqing Key Laboratory for the Prevention and Treatment of Major Blinding Eye Diseases, No.1, Youyi Road, Yuzhong District, Chongqing, 400016 China; 2 Department of Clinical Medicine, Chongqing Medical University, No.1, Yixueyuan Road, Yuzhong District, Chongqing, 400016 China

**Keywords:** Machine learning, Nomogram, Ocular hypertension, Rhegmatogenous retinal detachment, Silicone oils

## Abstract

**Introduction:**

This study aimed to develop predictive models for identifying risk factors of postoperative IOP elevation in patients with rhegmatogenous retinal detachment (RRD) following silicone oil (SO) tamponade.

**Methods:**

This retrospective study involved 533 RRD patients treated at the First Affiliated Hospital of Chongqing Medical University between September 1, 2024, and June 19, 2025. IOP was measured postoperatively on day 1, week 1, and months 1, 3, 6, and before SO removal using the non-contact tonometer Corvis Scheimpflug Technology (Corvis ST, OCULUS, Wetzlar, Germany) to acquire biomechanically corrected IOP (bIOP). Multiple machine learning (ML) models and one nomogram were constructed using demographic, clinical, and surgical factors.

**Results:**

Postoperatively, 57.8% of patients developed elevated intraocular pressure (IOP), with 81.5% occurring within two weeks. A Stepwise GLM [forward] + SVM model based on clinical features outperformed the Nomogram (AUC 0.761 vs. 0.729). The top five predictors were axial length (AL), age, white-to-white corneal diameter (WTW), proliferative vitreoretinopathy (PVR), and central corneal thickness (CCT).

**Conclusion:**

In RRD patients receiving silicone oil tamponade, AL, age, WTW, PVR, and CCT were closely associated with postoperative IOP elevation. The ML models outperformed nomogram in accuracy and clinical interpretability, offering a reliable tool utilizing bIOP for early IOP risk assessment and postoperative management in RRD patients. Their integration of anatomical and biometric features supports real-world clinical deployment and individualized care planning.

**Supplementary Information:**

The online version contains supplementary material available at 10.1186/s12886-026-04728-6.

## Introduction

Rhegmatogenous retinal detachment (RRD) is a severe blinding ocular disease characterized by liquefied vitreous entering the subretinal space through retinal breaks, resulting in the separation of the retinal neuroepithelium from the retinal pigment epithelium [1]. Pars plana vitrectomy (PPV) combined with intraocular tamponade has become the standard surgical approach for managing RRD [2]. Silicone oil (SO) is a hydrophobic polymer compound composed of silicon-oxygen bonds, demonstrating stability, transparency, non-toxicity, and excellent biocompatibility in the intraocular environment. Its favorable buoyancy and high surface tension help maintain the anatomical relationship between the retinal neuroepithelium and the retinal pigment epithelium, making it a widely used tamponade agent in vitreoretinal surgery [3, 4].

The safety and efficacy of SO have been extensively demonstrated in long-term clinical use. However, concerns persist regarding its potential side effects, including elevated intraocular pressure (IOP), cataracts, corneal changes, optic nerve damage, and SO emulsification [5, 6]. Among these, elevated IOP is one of the most frequent complications. Literature reports that the incidence of SO-related IOP elevation ranges from 2.2% to 56.0% [7], in some cases is significantly associated with visual prognosis [8]. The underlying pathological mechanisms of SO-related IOP elevation are multifactorial and not fully understood, potentially involving both early and late postoperative inflammation, ciliary body edema, pupillary block, and SO emulsification [9, 10].

Retinal detachment (RD) is a risk factor for elevated IOP following PPV combined with SO tamponade, with a higher incidence than other vitreoretinal diseases, such as macular holes [11]. This phenomenon may be associated with the frequent use of laser photocoagulation in RD patients during surgery, which can induce choroidal congestion and a stronger postoperative inflammatory response, thereby increasing the risk of IOP elevation [12]. A comprehensive understanding of the risk factors for elevated IOP in RRD patients following PPV with SO tamponade can effectively prevent and reduce its incidence, thereby minimizing its impact on visual function. Recent studies have applied data-driven methods to predict postoperative IOP elevation after silicone oil tamponade. However, these models often included heterogeneous vitreoretinal diseases or relied on conventional tonometry with lower IOP thresholds (> 21 mmHg), limiting their applicability to RRD patients and clinically significant ocular hypertension [11, 13]. To address this gap, the present study focused specifically on patients with rhegmatogenous retinal detachment undergoing pars plana vitrectomy with silicone oil tamponade. Unlike previous studies that included heterogeneous vitreoretinal conditions or relied on conventional tonometry with relatively low IOP thresholds, we aimed to characterize clinically meaningful early postoperative ocular hypertension using biomechanically corrected intraocular pressure (bIOP). Furthermore, rather than applying machine learning as an end in itself, we integrated anatomical, biometric, and surgical parameters to develop interpretable prediction tools intended for early risk stratification and individualized postoperative management in real-world clinical practice.

## Methods

### Study design and participants

This single-center, retrospective observational study was performed at the First Affiliated Hospital of Chongqing Medical University, China. We reviewed the medical records of patients who were diagnosed with RRD and underwent PPV combined with SO (Arciolane 5500 cSt SO) tamponade between September 1, 2024, and June 19, 2025. The study adhered to the tenets of the Declaration of Helsinki and was approved by the institutional review board, with the requirement for informed consent waived due to its retrospective nature. All surgeries were performed by experienced vitreoretinal specialists following a standardized protocol. A complete fluid-air exchange was conducted prior to silicone oil injection to ensure a uniform fill of the vitreous cavity. IOP was assessed by digital palpation at the conclusion of surgery and meticulously calibrated to a normal level (Tn), thereby standardizing the final fill volume and minimizing the risk of postoperative IOP fluctuations attributable to overfilling or underfilling.

- **Inclusion Criteria**: (1) Diagnosis of RRD; (2) Age between 18 and 85 years; (3) Preoperative IOP < 21 mmHg in both eyes, with normal anterior chamber anatomy and no history or family history of glaucoma.
- **Exclusion Criteria**: (1) Congenital ocular anomalies or history of ocular trauma in the study eye; (2) Significant media opacities (e.g., corneal scar, leukoma) precluding adequate fundus view; (3) Pre-existing glaucoma or other ocular diseases; (4) History of other vitreoretinal diseases; (5) Systemic diseases such as autoimmune disorders or long-term corticosteroid use. (6) Documented poor adherence to the standardized postoperative facedown and non-supine positioning protocol.(7) occurrence of complications directly attributable to improper silicone oil fill volume (e.g., persistent hypotony or overfill-induced IOP spike) as documented in the medical records.

A preliminary analysis indicated an incidence of postoperative IOP elevation of 55%. According to the Events Per Variable (EPV) criterion of 10 for logistic regression and assuming at least 20 predictor variables [14], a minimum sample size of 364 patients was required to minimize overfitting and ensure robust model development.

### Data collection and measurements

For each participant, one eye was randomly selected if both were eligible. Data were collected on:

- **Demographics and Baseline Characteristics**: Age, sex, laterality of the affected eye, best-corrected visual acuity (BCVA), refractive status, lens status, systemic comorbidities, and history of ocular surgery.
- **Preoperative Laboratory Tests**: Complete blood count (CBC) results.
- **Clinical Ocular Features**: Duration of retinal detachment, number, size, and location of retinal breaks, extent of detachment, and proliferative vitreoretinopathy (PVR) grade according to the Retina Society classification (1983) [15].
- **Ocular Biometry**: Axial length (AL) was measured using the IOL Master 700 (Carl Zeiss Meditec) or B-scan ultrasound when necessary. Anterior segment parameters, including central corneal thickness (CCT), white-to-white distance (WTW), anterior chamber depth (ACD), and lens thickness (LT), were obtained using the IOL Master 700.
- **Surgical Factors**: Operative time and concomitant cataract surgery.
- **Postoperative Outcomes**: Postoperative IOP was measured using the non-contact tonometer Corvis Scheimpflug Technology (Corvis ST, OCULUS, Wetzlar, Germany). Unlike conventional tonometry, the Corvis ST accounts for individual corneal biomechanical properties to provide a bIOP, which is largely independent of CCT and thus provides a more accurate reflection of true IOP [16]. Measurements were taken at postoperative day 1, week 1, and months 1, 3, and 6, as well as prior to SO removal. IOP elevation was defined as a bIOP value ≥ 25 mmHg.The extent of retinal detachment was quantified from preoperative wide-field fundus photographs (Zeiss CLARUS 500) using ImageJ software, with the fundus divided into eight regions for zonal analysis.

All image analyses were performed by masked ophthalmologists who were blinded to the patients’ IOP data and group assignments.

### Perioperative medication and IOP management

A standardized perioperative medication protocol was followed for all patients. Preoperatively, levofloxacin 0.5% eye drops were applied to the operative eye four times daily for 3 days for infection prophylaxis. Postoperatively, a fixed combination therapy was initiated immediately and continued for up to 2 weeks unless complications required adjustment. The core regimen and dosing schedule were consistent across patients. This regimen included: (1) levofloxacin 0.5% eye drops, (2) pranoprofen 0.1% eye drops, (3) polyethylene glycol 0.4% eye drops, (4) tobramycin 0.3% plus dexamethasone 0.1% eye drops (all four times daily), and (5) tobramycin 0.3% plus dexamethasone 0.1% eye ointment (once nightly at bedtime).

Patients who developed elevated IOP were managed by the operating surgeon based on measured IOP levels. Initial treatment included topical IOP-lowering medications, such as brimonidine, brinzolamide, timolol, and travoprost. If IOP remained uncontrolled, further interventions were performed as clinically indicated, including anterior chamber paracentesis, silicone oil removal, trabeculectomy, or glaucoma drainage device implantation.

### Handling of missing data

Missing data were present for several variables: white blood cell count and its derivatives (4.30%), ACD (10.13%), WTW (14.82%), and CCT (14.63%). These missing values were handled using multiple imputation techniques [17].

### Statistical analysis and model development

#### Predictor selection and nomogram prediction model development

Predictors were selected using the Least Absolute Shrinkage and Selection Operator (LASSO) regression method. The selected variables were then incorporated into a multivariable logistic regression model to build a predictive nomogram. The model underwent internal validation with 1000 bootstrap resamples. Model performance was assessed by the receiver operating characteristic (ROC) curve and area under the curve (AUC). Calibration was evaluated using calibration plots, and clinical utility was appraised via decision curve analysis (DCA) and clinical impact curves (CIC).

#### Machine learning model development

The dataset was stratified and randomly split into a training set (70%, *n* = 373) and a test set (30%, *n* = 160). To ensure robustness, five distinct feature selection methods (LASSO, Elastic Net, and forward, backward, and stepwise selection) were applied in parallel. Each resulting feature subset was used to train nine different classification algorithms (including Support Vector Machine, Random Forest, and XGBoost), generating 45 candidate models. Hyperparameters were optimized via grid search with 5-fold cross-validation on the training set. The final models were evaluated on the held-out test set. High-confidence models were selected based on superior AUC and minimal performance discrepancy between the training and test sets. SHapley Additive exPlanations (SHAP) analysis was employed to interpret the best-performing model.

All analyses were performed using R software (version 4.5.0), IBM SPSS Statistics (version 26.0), and Python (version 3.10.4). Continuous variables are presented as mean ± standard deviation, and categorical variables as frequencies (percentages). Group comparisons were made using Student’s t-test, Mann-Whitney U test, Chi-square test, or Fisher’s exact test, as appropriate. A two-sided p-value < 0.05 was considered statistically significant.

## Results

### Baseline demographic and clinical characteristics

**Fig. 1:**
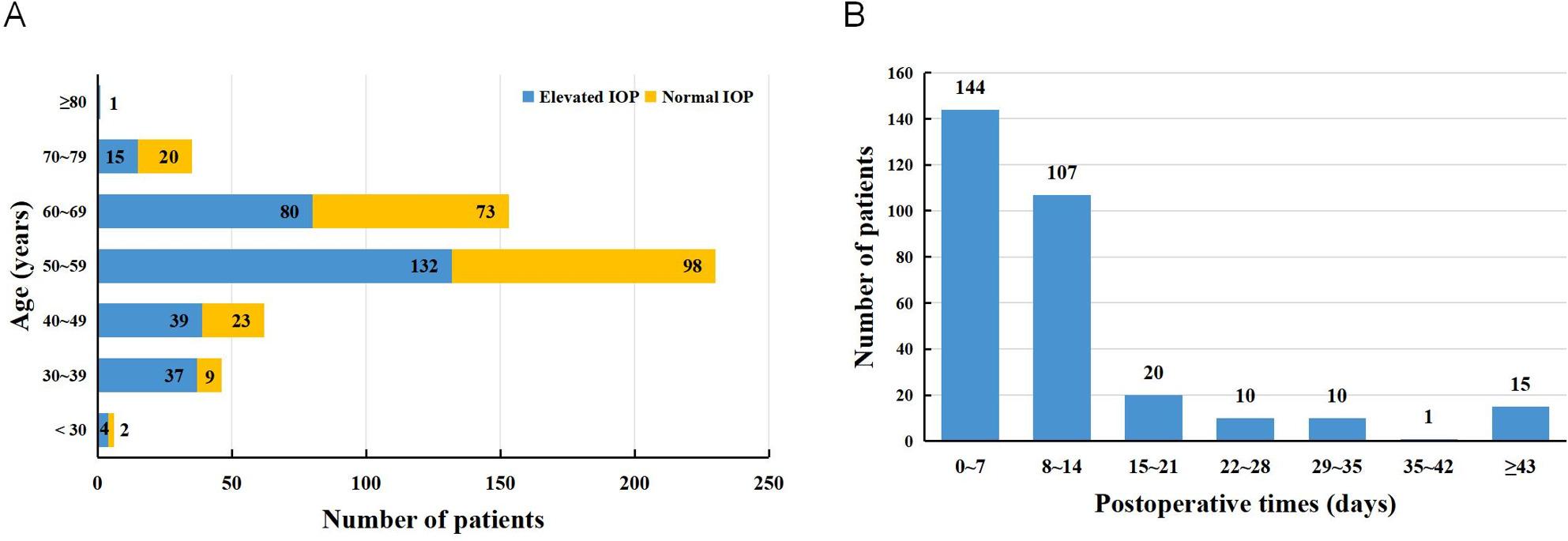
Postoperative IOP elevation according to Time and age stratification[A] Age-stratified distribution of postoperative intraocular pressure elevation, demonstrating a higher proportion of early IOP elevation among younger patients. [B] Temporal distribution of patients with elevated Intraocular Pressure after surgery

A total of 533 patients diagnosed with RRD underwent PPV with SO tamponade. The mean age was 53.82 ± 10.21 years, including 287 males (53.85%) and 246 females (46.15%). No significant differences in baseline characteristics were found among the overall, training, and validation cohorts (Table 1). In most cases, postoperative IOP elevation was transient, peaking in the early postoperative period and subsequently stabilizing or decreasing with routine postoperative management; notably, none of the patients met diagnostic criteria for secondary glaucoma during the follow-up period. When stratified by age, younger patients demonstrated a higher proportion of postoperative IOP elevation compared with older patients (Fig. 1A). During follow-up, 308 patients (57.79%) developed elevated IOP, with 251 cases (81.49%) occurring within the first two postoperative weeks (Fig. 1B).

**Table 1:** Demographic characteristics of the patients with RRD

Characteristic		ALL (*n* = 533)	Elevated IOP (*n* = 308)	Normal IOP (*n* = 255)	*P* value
Age		55.33 (10.21)	53.83 (10.51)	57.38 (9.41)	**< 0.001**
Sex	Male	246 (46.2%)	126 (40.9%)	120 (53.3%)	**0.006**
	Female	287 (53.8%)	182 (59.1%)	105 (46.7%)	
Diabetes Mellitus	No	504 (94.6%)	292 (94.8%)	212 (94.2%)	0.921
	Yes	29 (5.4%)	16 (5.2%)	13 (5.8%)	
Hypertension	No	447 (83.9%)	263 (85.4%)	184 (81.8%)	0.317
	Yes	86 (16.1%)	45 (14.6%)	41 (18.2%)	
Surgical Eye	OD	291 (54.6%)	161 (52.3%)	130 (57.8%)	0.241
	OS	242 (45.4%)	147 (47.7%)	95 (42.2%)	
Preoperative Visual Acuity (logMAR)		1.56 (0.77)	1.56 (0.76)	1.57 (0.78)	0.871
Ophthalmic Surgery History	No	445 (83.5%)	254 (82.5%)	191 (84.9%)	0.532
	Yes	88 (16.5%)	54 (17.5%)	34 (15.1%)	
Ophthalmic Disease History	No	475 (89.1%)	276 (89.6%)	199 (88.4%)	0.775
	Yes	58 (10.9%)	32 (10.4%)	26 (11.6%)	
Associated Vitreous Hemorrhage	No	498 (93.4%)	286 (92.9%)	212 (94.2%)	0.652
	Yes	35 (6.6%)	22 (7.1%)	13 (5.8%)	
Number of Retinal Detachments		1.05 (0.22)	1.06 (0.23)	1.04 (0.2)	0.338
Duration of retinal detachment		27.34 (64.57)	28.21 (63.09)	26.15 (66.68)	0.713
Number of Holes		1.7 (1.15)	1.76 (1.21)	1.61 (1.06)	0.219
Largest Retinal Hole		2.29 (1.86)	2.2 (1.71)	2.41 (2.05)	0.454
Extent of Retinal Detachment (%)		53.19 (26.05)	55.27 (27.75)	50.34 (23.29)	0.154
Macular-involved	Yes	377 (70.7%)	215 (69.8%)	162 (72%)	0.650
	No	156 (29.3%)	93 (30.2%)	63 (28%)	
Axial Length		25.94 (2.6)	26.34 (2.65)	25.39 (2.43)	**< 0.001**
PVR	A	54 (10.1%)	19 (6.2%)	35 (15.6%)	
	B	217 (40.7%)	126 (40.9%)	91 (40.4%)	
	C	211 (39.6%)	124 (40.3%)	87 (38.7%)	
	D	51 (9.6%)	39 (12.7%)	12 (5.3%)	
Associated Choroidal Detachment	No	492 (92.3%)	284 (92.2%)	208 (92.4%)	1.000
	Yes	41 (7.7%)	24 (7.8%)	17 (7.6%)	
WBC		6.29 (1.72)	6.45 (1.71)	6.08 (1.73)	**0.003**
NEUT%		63.21 (9.03)	63.34 (9.15)	63.03 (8.89)	0.833
LYM%		27.3 (7.72)	27.19 (7.87)	27.45 (7.52)	0.624
MONO%		6.92 (2.05)	6.79 (1.99)	7.09 (2.12)	0.096
EO%		2 (1.83)	2.08 (2.04)	1.89 (1.49)	0.765
BASO%		0.55 (0.28)	0.56 (0.3)	0.53 (0.26)	0.375
ACD (mm)		3.37 (0.58)	3.41 (0.57)	3.31 (0.6)	**0.022**
LT (mm)		3.71 (1.53)	3.63 (1.56)	3.83 (1.48)	**0.023**
WTW (mm)		11.77 (0.46)	11.76 (0.48)	11.79 (0.42)	**0.221**
CCT (µm)		534.97 (34.52)	541.24 (34.11)	526.39 (33.27)	**< 0.001**
Operation Time (min)		60.29 (19.79)	59.48 (18.81)	61.4 (21.05)	0.360
Combined Cataract Surgery	No	476 (89.3%)	273 (88.6%)	203 (90.2%)	0.658
	Yes	57 (10.7%)	35 (11.4%)	22 (9.8%)	

Note: Categorical variables are presented as n (%), and continuous variables are presented as mean (SD)

### Feature selection and models development

#### Development of the nomogram prediction model

LASSO regression was first applied to identify potential predictors while minimizing overfitting and improving model stability (Fig. S2). Based on the LASSO results, 22 candidate predictors were subsequently included in a multivariable logistic regression analysis (Table 2). This analysis ultimately identified seven independent predictors: age, sex, axial length of the operated eye, PVR grade, WTW, CCT, and surgical time.

**Fig. 2:**
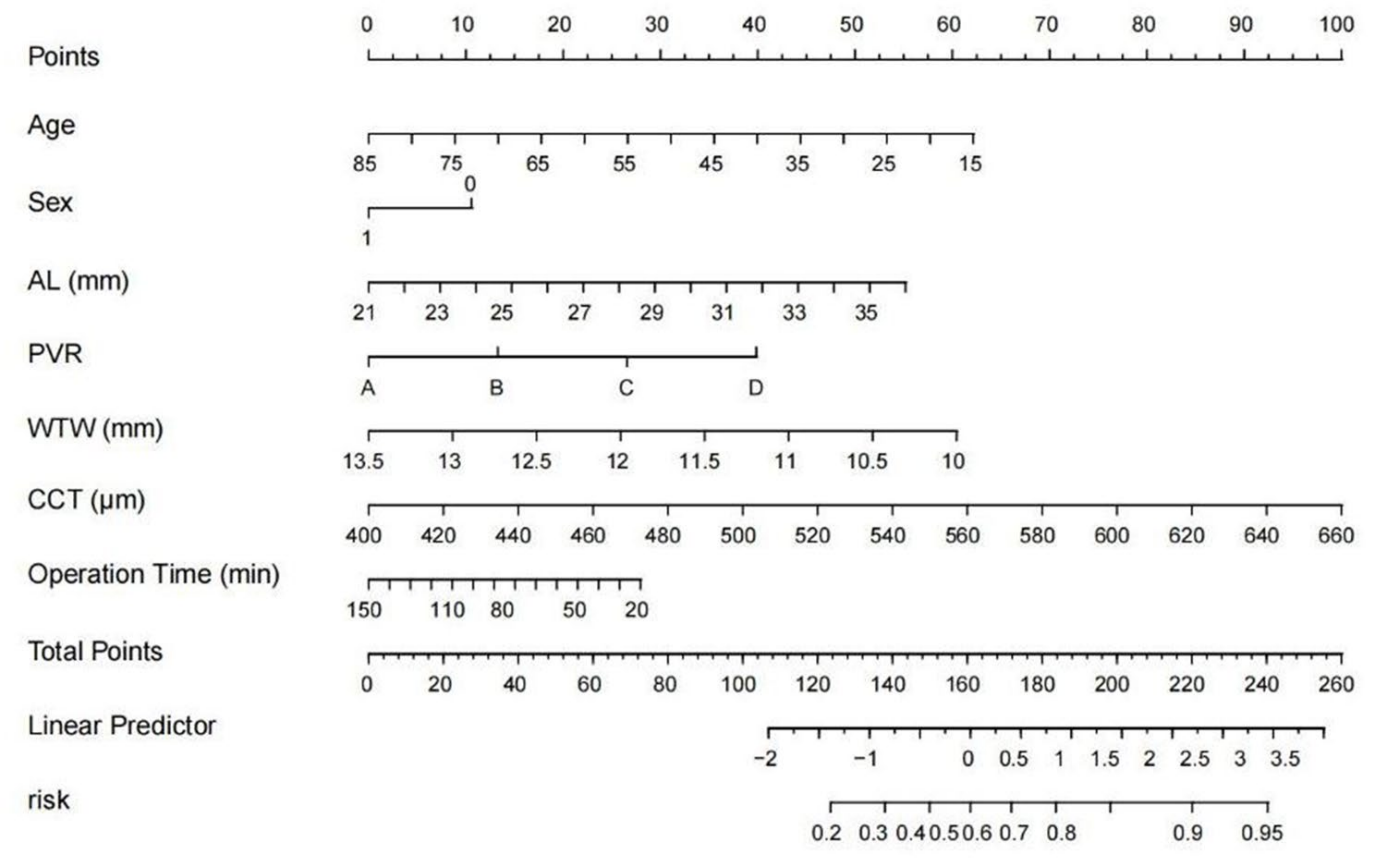
Nomogram to predict the probability of IOP elevation in patients with RRD after PPV combined with SO tamponade. Note: AL (mm), axial length, measured in millimeters; PVR, proliferative vitreoretinopathy; WTW (mm), white-to-white distance, measured in millimeters; CCT (um), central corneal thickness, measured in micrometers

Based on the seven identified predictors, a nomogram was developed to estimate the risk of elevated IOP following PPV with SO tamponade in patients with RRD (Fig. 2). In the nomogram, each predictor is assigned a point value, and the total score is obtained by summing these values. The probability of elevated IOP is then determined by projecting a vertical line from the total score to the risk scale.

**Table 2:** Multivariable logistic regression of risk factor for High IOP after SO tamponade in patients with RRD

Variables	β	S.E.	OR (95% CI)	*P* value
Age	-0.037	0.012	0.96 (0.94–0.99)	**0.001**
Sex	-0.469	0.217	0.63 (0.41–0.96)	**0.030**
Hypertension	-0.198	0.274	0.82 (0.48–1.41)	0.471
Surgical Eye	0.335	0.199	1.40 (0.95–2.07)	0.092
Preoperative Visual Acuity (log MAR)	-0.153	0.137	0.86 (0.66–1.12)	0.262
Associated Vitreous Hemorrhage	0.334	0.409	1.40 (0.63–3.18)	0.414
Number of Retinal Detachments	0.318	0.475	1.38 (0.55–3.61)	0.503
Duration of retinal detachment	0.002	0.002	1.00 (1.00-1.01)	0.329
Largest Retinal Hole	-0.084	0.055	0.92 (0.82–1.02)	0.126
Extent of Retinal Detachment (%	0.005	0.005	1.00 (1.00-1.01)	0.299
Macular-involved	-0.208	0.234	0.81 (0.51–1.28)	0.374
Axial Length	0.135	0.044	1.14 (1.05–1.25)	**0.002**
PVR	0.477	0.14	1.61 (1.23–2.13)	**< 0.001**
WBC	0.049	0.063	1.05 (0.93–1.19)	0.431
MONO%	-0.085	0.051	0.92 (0.83–1.01)	0.094
EO%	0.047	0.068	1.05 (0.92–1.20)	0.482
BASO%	0.753	0.408	2.12 (0.96–4.79)	0.065
ACD (mm)	0.169	0.189	1.18 (0.82–1.72)	0.371
WTW (mm)	-0.745	0.243	0.47 (0.29–0.76)	**0.002**
CCT(µm)	0.015	0.003	1.02 (1.01–1.02)	**< 0.001**
Operation Time (min)	-0.012	0.005	0.99 (0.98-1.00)	**0.024**
Combined Cataract Surgery	0.428	0.356	1.53 (0.77–3.12)	0.229

**Fig. 3:**
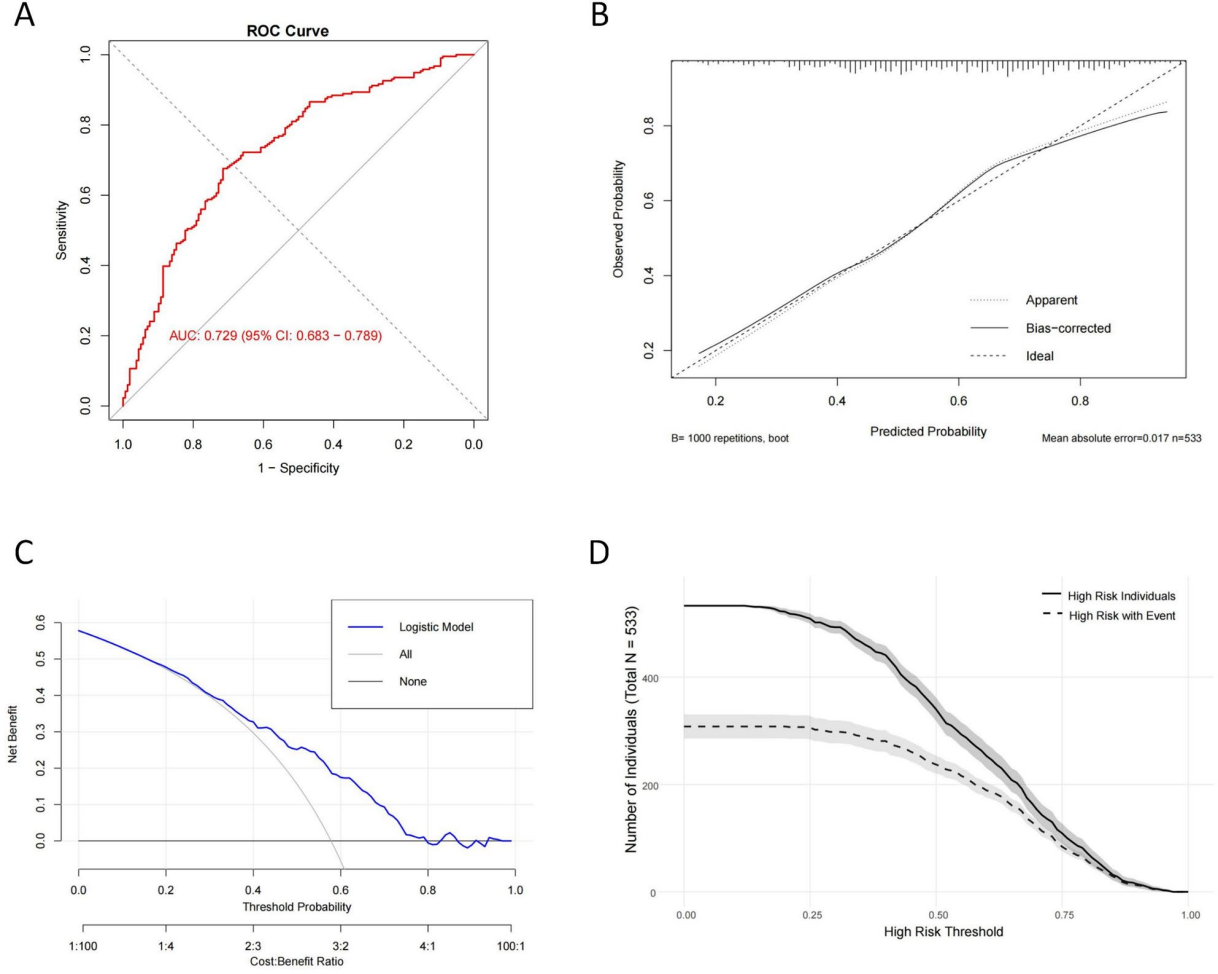
Performance evaluation of the nomogram model. (**A**) The receiver operating characteristic (ROC) curve of the nomogram. (**B**) Calibration curve of the nomogram. The x-axis represents the predicted probability of heterogeneous frailty trajectories derived from the model, while the y-axis indicates the observed incidence. (**C**) Decision curve analysis (DCA) of the nomogram. The y-axis represents the net benefit, and the x-axis represents threshold probabilities. (**D**) Clinical impact curve (CIC) of the nomogram. The black solid line indicates the number of individuals classified as high risk at each threshold, while the black dashed line indicates the number of true positives

The nomogram demonstrated good discrimination, with an AUC of 0.729 (Bootstrap 1000x: 95%CI = 0.683–0.789) (Fig. 3A). The calibration curve closely aligned with the ideal 45° line, with a mean absolute error of 0.017, indicating strong agreement between predicted and observed probabilities (Fig. 3B). DCA showed that the nomogram provided greater net benefit than the “Treat All” and “Treat None” strategies across a threshold probability range of 0.2 to 0.8, suggesting good clinical utility (Fig. 3C). CIC further supported the model’s utility, as the number of high-risk patients identified closely matched the number of true positive cases (Fig. 3D).

#### Development of the machine learning prediction model

**Fig. 4:**
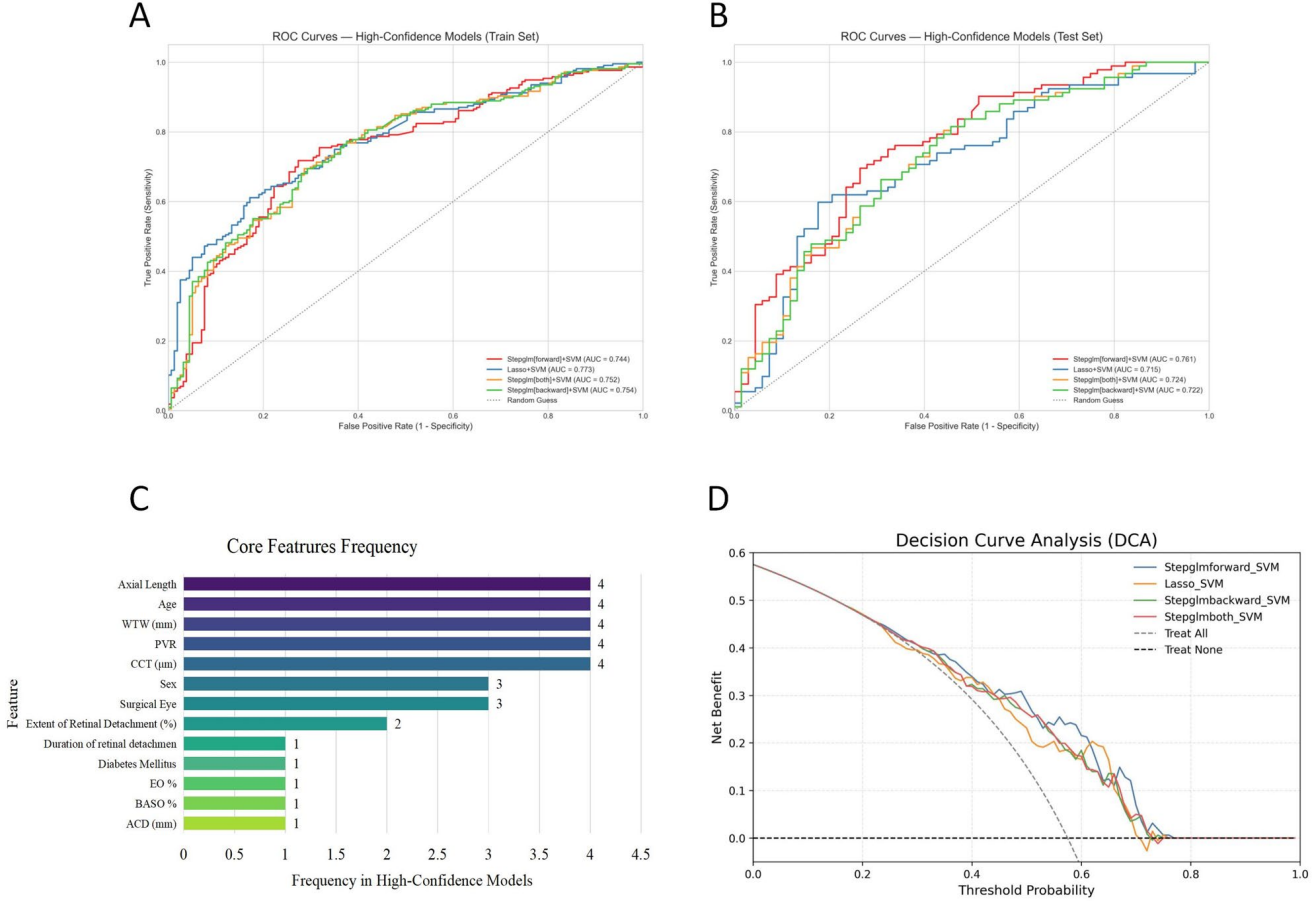
Machine learning model performance and feature analysis. (**A**) Training set ROC curves of four machine learning models. (**B**) Test set ROC curves of four machine learning models. (**C**) Frequency distribution of core features selected by the four machine learning models. (**D**) Decision curve analysis (DCA) curves of four machine learning models

A total of 45 machine learning model combinations were systematically developed and evaluated. After rigorous screening, four high- performing models were identified: Stepglm [forward] + SVM, Lasso + SVM, Stepglm [both] + SVM, and Stepglm [backward] + SVM. These models achieved AUC values ranging from 0.715 to 0.761 in the test set, with the Stepglm [forward] + SVM model showing the best performance (AUC = 0.761 in the test set and 0.744 in the training set), demonstrating good generalizability without overfitting (Table 3; Fig. 4A–B). The main predictive factors were AL, age, WTW, PVR, and CCT (Fig. 4C). DCA showed that, within a threshold probability of 0.2–0.8, the Stepglm [forward] + SVM model achieved a substantially higher net benefit than the “treat-all” and “treat-none” strategies (Fig. 4D).

**Table 3:** Comparison of classification performance across four machine learning models on train and test sets

Model	Dataset	AUC (95% CI)	Accuracy	Precision	Sensitivity	Specificity	F1
Stepglm (forward) + SVM	Train	0.744(0.693–0.794)	0.721	0.783	0.718	0.726	0.749
Stepglm (forward) + SVM	Test	0.761(0.681–0.834)	0.713	0.756	0.739	0.676	0.747
Lasso + SVM	Train	0.773(0.727–0.818)	0.700	0.752	0.718	0.675	0.735
Lasso + SVM	Test	0.715(0.634–0.797)	0.669	0.714	0.707	0.618	0.71
Stepglm (backward) + SVM	Train	0.754(0.703–0.802)	0.697	0.756	0.704	0.688	0.729
Stepglm (backward) + SVM	Test	0.722(0.643–0.801)	0.662	0.716	0.685	0.632	0.7
Stepglm (both) + SVM	Train	0.752(0.701-0.800)	0.700	0.76	0.704	0.694	0.731
Stepglm (both) + SVM	Test	0.724(0.645–0.803)	0.662	0.716	0.685	0.632	0.700

**Fig. 5:**
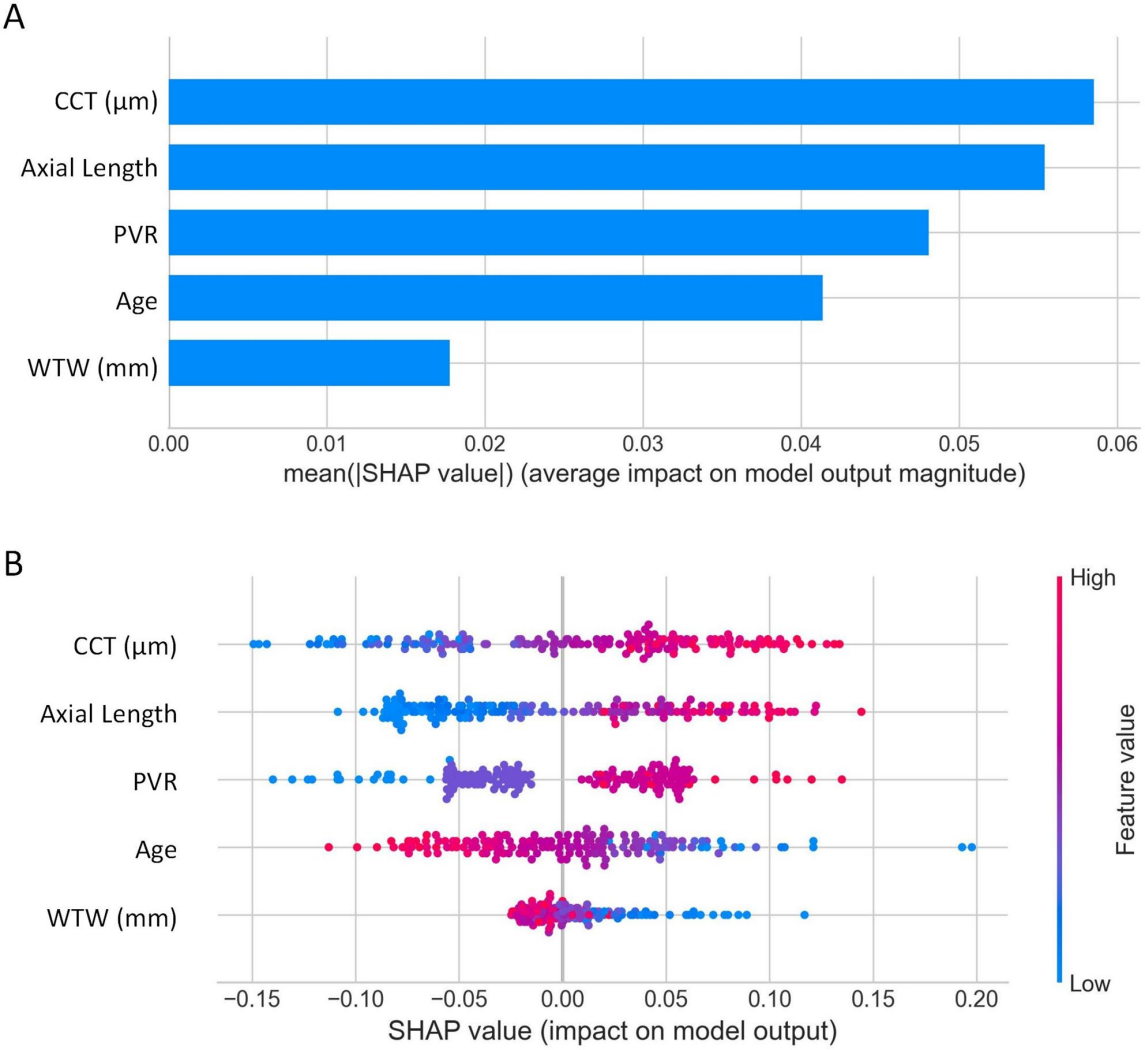
SHAP analysis of feature importance for the Stepglm [forward] + SVM Model. (**A**) SHAP mean absolute value bar plot of the Stepglm(forward) + SVM model. (**B**) SHAP beeswarm plot of the Stepglm(forward) + SVM model

To elucidate the internal decision-making process of the optimal Stepglm [forward] + SVM model, SHAP analysis was performed. The results indicated that CCT had the strongest effect on the model output, followed by AL, PVR, age, and WTW, consistent with the previously identified key predictors (Fig. 5A). Higher CCT, AL, and PVR values were associated with a greater likelihood of postoperative IOP elevation, whereas older age and smaller WTW were linked to lower risk (Fig. 5B).

## Discussion

Elevated IOP is one of the most common complications following PPV with SO tamponade for RRD and is closely associated with visual prognosis. Preventing and minimizing its occurrence is therefore of significant clinical importance. This study developed predictive models based on clinical characteristics of RRD patients, offering a practical tool for preoperative identification of high-risk individuals and for guiding personalized management strategies.

In this study, postoperative IOP elevation occurred in 57.79% of patients, with the majority of cases (81.49%) developing within the first two postoperative weeks. Both the nomogram and the machine learning–based models demonstrated acceptable performance in predicting postoperative IOP elevation using routinely available clinical features. Among the evaluated approaches, the Stepglm (forward) + SVM model achieved the highest discriminative performance, with axial length, age, white-to-white distance, proliferative vitreoretinopathy, and central corneal thickness emerging as the most influential predictors. Decision curve analysis further suggested that these models may provide clinical benefit across a wide range of threshold probabilities (20–80%). Although the machine learning models showed slightly higher AUC values than the nomogram, the magnitude of this difference was modest. Importantly, the nomogram offers greater transparency and ease of implementation in daily clinical practice, whereas machine learning models provide enhanced flexibility in capturing complex, nonlinear relationships among predictors. These approaches should therefore be viewed as complementary rather than competing tools for postoperative IOP risk assessment, allowing clinicians to balance interpretability and predictive performance according to specific clinical contexts.

It is noteworthy that postoperative IOP in this study was assessed using bIOP derived from Corvis ST. Rather than serving as a replacement for conventional tonometry, bIOP was used as a methodological refinement to reduce the potential influence of corneal biomechanical properties on IOP measurement [16]. This approach may allow for a more consistent assessment of postoperative IOP changes, particularly when comparing patients with varying corneal characteristics.

Silicone oil emulsification—a process involving the breakdown of oil into microscopic droplets—is a time-dependent phenomenon that typically manifests over months to years [18]. Therefore, the early postoperative IOP elevation observed in this study is more plausibly attributed to immediate factors. Younger age was identified as a risk factor, likely due to stronger postoperative inflammatory responses [11, 12, 19]. PPV can induce inflammation and disrupt the blood-ocular barrier [20]. Obstruction of the trabecular meshwork by inflammatory cells or fibrin, together with edema of the trabecular meshwork and ciliary body, can impede aqueous humor outflow, resulting in elevated IOP [19]. Compared to elderly patients, younger individuals exhibit stronger immune and reparative capacities, resulting in more intense postoperative inflammatory responses [21]. Therefore, age acts as a protective factor against postoperative IOP elevation. Moreover, disruption of aqueous humor dynamics following silicone oil tamponade [22], as well as postoperative hydrogen peroxide (H₂O₂) and free radical-induced damage to the trabecular meshwork [23], have been implicated in early postoperative IOP elevation.

Previous studies have established associations between ocular biometric parameters—such as CCT, ACD, LT, and WTW are associated with IOP [24, 25]. Research by Katsimpris [26] indicates that CCT may serve as a potential risk factor for primary open-angle glaucoma (POAG). A multicenter study involving 7,983 participants demonstrated a positive correlation between WTW and ACD [27], with ACD acting as a surrogate biomarker for mechanical obstruction of the aqueous humor outflow pathway, which is significantly associated with IOP [28]. Consequently, WTW may also be a potential influencing factor for IOP. Axial elongation may lead to thinning of the sclera, choroid, and trabecular meshwork, impairing aqueous drainage, while SO buoyancy can weaken the zonular fibers and cause anterior lens displacement, further impeding outflow [29, 30]. Our findings suggest that AL, CCT, and WTW are closely related to postoperative IOP elevation, underscoring the clinical relevance of ocular biometry under specific pathological conditions such as RRD. Further studies are warranted to clarify postoperative changes in ocular parameters and their relationship with IOP after PPV and SO tamponade.

Lens status has also been reported to influence postoperative IOP elevation, with aphakia identified as a risk factor [31–33]. One possible mechanism involves intraocular oxygen diffusion [34], which may damage the trabecular meshwork and reduce aqueous outflow [35]. The crystalline lens contributes to oxygen metabolism [36], thereby mitigating oxidative stress and acting as a physical barrier that limits anterior chamber oxygen exposure. However, no significant association between lens status and postoperative IOP elevation was found in this study, likely due to the small proportion of aphakic patients (2%).

All patients received a standardized short-term postoperative topical regimen including dexamethasone (≤ 2 weeks). Although corticosteroids are known to elevate IOP [37], the majority of IOP elevation events (81.5%) occurred within the first two postoperative weeks, coinciding with the prophylactic steroid period. If steroids were the primary driver, one would expect delayed or persistent IOP elevation after discontinuation, which was not observed. These findings suggest that early postoperative IOP elevation is primarily driven by patient-specific anatomical (e.g., axial length, CCT) and pathological factors (PVR grade) rather than short-term steroid use. The retrospective nature of this study precludes definitive causal inference; future prospective studies with detailed medication records and longer follow-up are warranted to confirm these observations.

Previous studies have mainly focused on the association between PVR grading and recurrent retinal detachment, with limited evidence linking PVR to postoperative IOP elevation. In this study, PVR grade was identified as a significant risk factor, suggesting that PVR-related inflammatory and fibrotic processes may disrupt IOP regulation. PVR formation involves inflammation, cellular proliferation, and epiretinal membrane development [38]. Elevated levels of inflammatory cytokines such as VEGF, IL-6, FGF2, and G-CSF have been reported in patients with postoperative IOP elevation and correlate positively with preoperative PVR grade [39]. These mediators may alter aqueous humor dynamics by promoting inflammation and modifying the anterior segment microenvironment. Moreover, more complex surgeries required for high-grade PVR may further exacerbate postoperative inflammation, contributing to IOP elevation.

Interestingly, 42.2% of patients did not experience postoperative IOP elevation. Potential protective factors may include older age, shorter axial length, lower CCT, absence of high-grade PVR, and favorable anterior chamber anatomy (e.g., deeper ACD or smaller WTW), which reduce susceptibility to trabecular outflow obstruction and postoperative inflammatory effects [40]. These findings underscore that postoperative IOP elevation is not universal and highlight the importance of individualized risk stratification. From a clinical perspective, the proposed prediction tools may assist ophthalmologists in identifying patients at higher risk of early postoperative ocular hypertension following silicone oil tamponade. Patients classified as high risk could benefit from closer IOP monitoring during the early postoperative period, earlier initiation of anti-glaucoma therapy, or more individualized follow-up schedules. By integrating readily available preoperative and intraoperative parameters, the models are designed to support clinical decision-making without adding procedural burden to routine clinical practice.

This study has several limitations: (1) The model was developed and validated based on data from a single tertiary center. While this single-center design ensured standardized surgical protocols, consistent postoperative management, and homogeneous data acquisition—which are crucial for minimizing confounding factors during initial model building—it inevitably limits the immediate generalizability of our findings. External validation using multi-center, prospective cohorts is necessary to confirm the model’s robustness and transportability across diverse clinical settings. (2) The retrospective nature of the study precludes the establishment of causal inferences. Future directions will include proactive collaboration with external institutions to perform independent validation and, ideally, prospective studies to further refine the model and assess its impact on clinical decision-making. (3) The use of Corvis ST for bIOP measurement may limit immediate generalizability, as this device is not universally available in all clinical settings.

## Conclusion

Ocular hypertension is a significant sight-affecting complication following pars plana vitrectomy with silicone oil tamponade for RRD. This study successfully developed and validated a user-friendly clinical prediction tool designed to identify high-risk patients preoperatively. By integrating key clinical parameters such as AL, age, WTW, PVR grade, and CCT, the tool enables individualized risk stratification. Validation results confirmed its robust discriminative ability and clinical utility, demonstrating optimal net benefit across a wide range of decision thresholds. This tool provides surgeons with valuable decision support, facilitating closer monitoring and early intervention for high-risk individuals to ultimately improve surgical outcomes.

## Supplementary Information

Below is the link to the electronic supplementary material.

Supplementary Material 1

## Data Availability

The data that support the findings of this study are not publicly available due to privacy and ethical restrictions related to patient confidentiality. However, they are available from the corresponding author (Prof. Hui Peng, email: pengh9@sina.com) upon reasonable request. Data requests will be reviewed and require approval from the Institutional Review Board of the First Affiliated Hospital of Chongqing Medical University.
