# Supplementary material for: A clinical prediction tool for ocular hypertension following silicone oil tamponade in rhegmatogenous retinal detachment
Source: BMC Ophthalmol. 2026 Mar 14;26:201. doi: 10.1186/s12886-026-04728-6 (PMC13101243; doi:10.1186/s12886-026-04728-6)
Supplement: Supplementary file 1 — Supplementary Material 1 [file 12886_2026_4728_MOESM1_ESM.doc]

**Supplementary material**

**Figure S1**. Illustration of Segmentation Zones in Fundus Photography.

**Figure S2.** LASSO Regression: Coefficient Profiles and Optimal λ Selection

**Table S1.** Baseline characteristics of the test and training groups


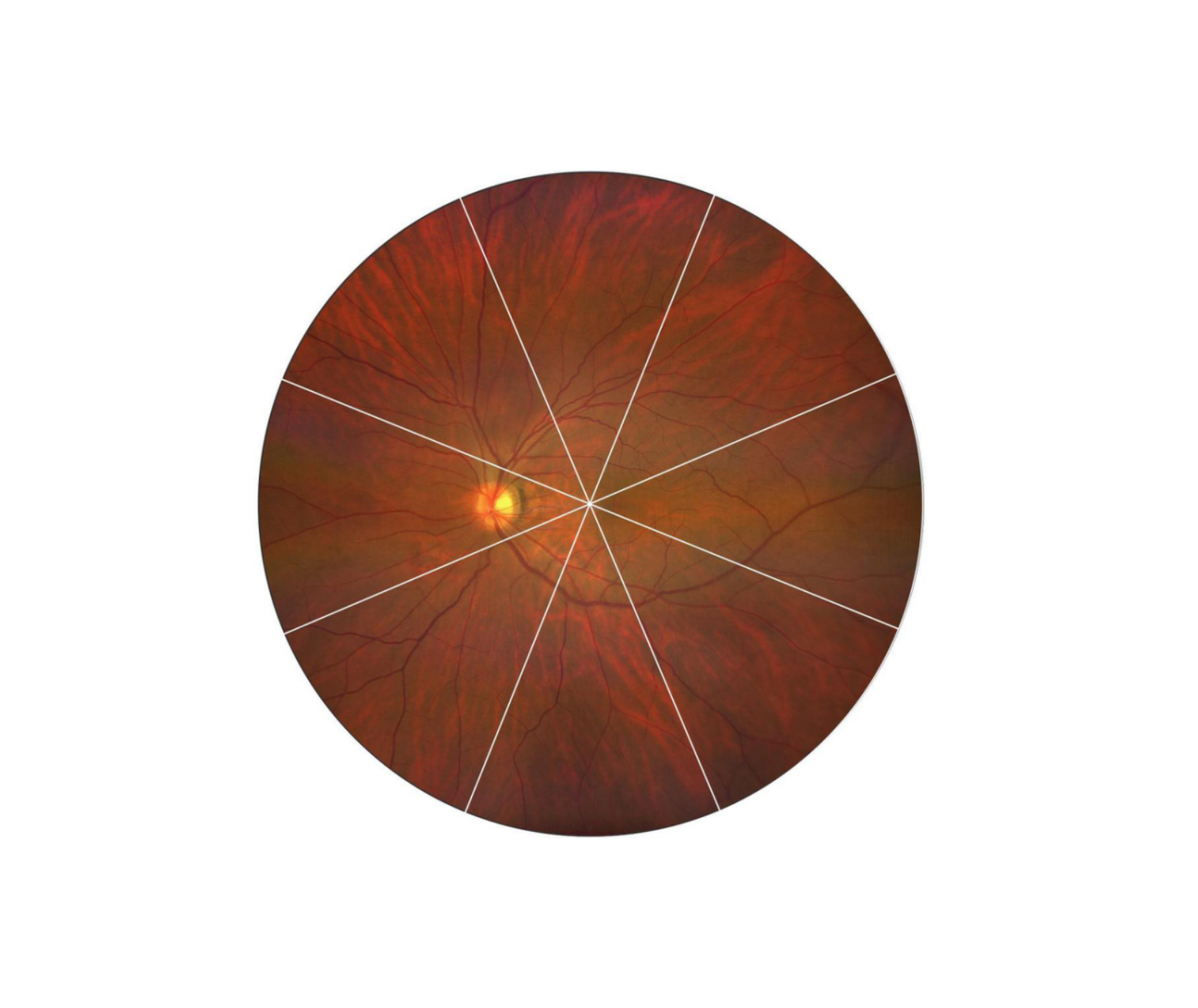


**Figure S1**. Illustration of Segmentation Zones in Fundus Photography.


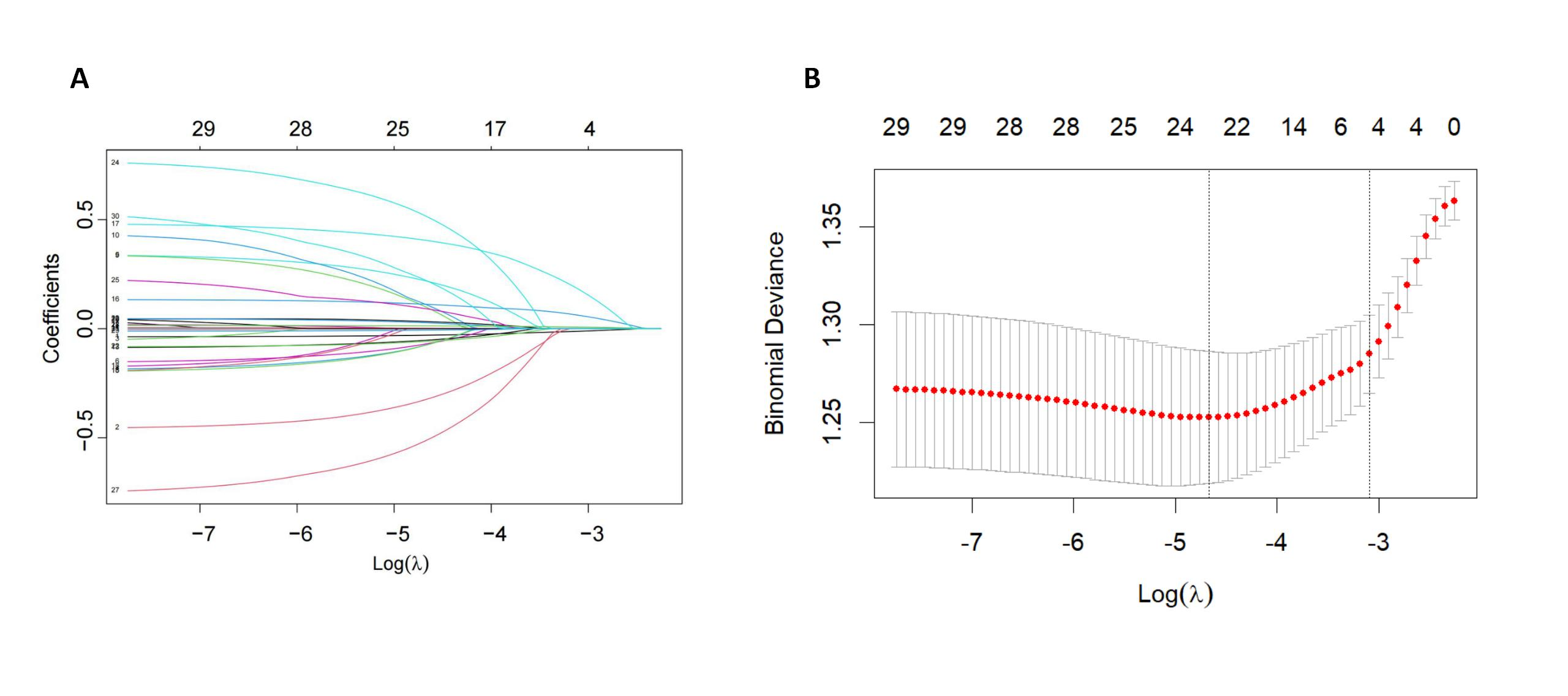


**Figure S2.** LASSO Regression: Coefficient Profiles and Optimal λ Selection.

[A] LASSO coefficient profiles of 30 clinical features. [B] The optimal parameter [λ] selection in the LASSO model employed 10-fold cross-validation. The optimal values of λ are represented by dotted vertical lines. Among these values, λ=0.00935, was selected as the optimal choice, corresponding to a model with 22 features having non -zero coefficients.

| **Table S1. Baseline characteristics of the test and training groups** | | | | |
| --- | --- | --- | --- | --- |
| **Characteristic** | | Test Group | Train Group | P value |
| **High IOP** | **No** | 68 (42.5%) | 157 (42.1%) | 1.000 |
|  | **Yes** | 92 (57.5%) | 216 (57.9%) |  |
| **Age** |  | 55.41 (10.27) | 55.29 (10.19) | 0.974 |
| **Sex** | **Male** | 67 (41.9%) | 179 (48%) | 0.229 |
|  | **Female** | 93 (58.1%) | 194 (52%) |  |
| **Diabetes Mellitus** | **No** | 154 (96.2%) | 350 (93.8%) | 0.358 |
|  | **Yes** | 6 (3.8%) | 23 (6.2%) |  |
| **Hypertension** | **No** | 137 (85.6%) | 310 (83.1%) | 0.552 |
|  | **Yes** | 23 (14.4%) | 63 (16.9%) |  |
| **Surgical Eye** | **OD** | 94 (58.8%) | 197 (52.8%) | 0.243 |
|  | **OS** | 66 (41.2%) | 176 (47.2%) |  |
| **Preoperative Visual Acuity (logMAR)** |  | 1.57 (0.73) | 1.56 (0.78) | 0.746 |
| **Ophthalmic Surgery History** | **No** | 135 (84.4%) | 310 (83.1%) | 0.816 |
|  | **Yes** | 25 (15.6%) | 63 (16.9%) |  |
| **Ophthalmic Disease History** | **No** | 145 (90.6%) | 330 (88.5%) | 0.562 |
|  | **Yes** | 15 (9.4%) | 43 (11.5%) |  |
| **Associated Vitreous Hemorrhage** | **No** | 148 (92.5%) | 350 (93.8%) | 0.705 |
|  | **Yes** | 12 (7.5%) | 23 (6.2%) |  |
| **Number of Retinal Detachments** |  | 1.04 (0.19) | 1.06 (0.23) | 0.365 |
| **Duration of retinal detachmen** |  | 25.73 (67.3) | 28.03 (63.45) | 0.995 |
| **Number of Holes** |  | 1.55 (1) | 1.76 (1.21) | 0.081 |
| **Largest Retinal Hole** |  | 2.2 (1.75) | 2.32 (1.91) | 0.553 |
| **Extent of Retinal Detachment (%)** |  | 52.35 (25.47) | 53.55 (26.33) | 0.708 |
| **Macular-involved** | **Yes** | 114 (71.2%) | 263 (70.5%) | 0.945 |
|  | **No** | 46 (28.8%) | 110 (29.5%) |  |
| **Axial Length** |  | 25.91 (2.67) | 25.95 (2.57) | 0.632 |
| **PVR** | **A** | 13 (8.1%) | 38 (10.2%) | 0.812 |
|  | **B** | 63 (39.4%) | 154 (41.3%) |  |
|  | **C** | 67 (41.9%) | 144 (38.6%) |  |
|  | **D** | 17 (10.6%) | 37 (9.9%) |  |
| **Associated Choroidal Detachment** | **No** | 149 (93.1%) | 343 (92%) | 0.775 |
|  | **Yes** | 11 (6.9%) | 30 (8%) |  |
| **WBC** |  | 6.14 (1.59) | 6.36 (1.77) | 0.179 |
| **NEUT%** |  | 63.4 (8.59) | 63.13 (9.23) | 0.782 |
| **LYM%** |  | 27.31 (7.36) | 27.29 (7.88) | 0.910 |
| **MONO%** |  | 6.93 (1.99) | 6.92 (2.07) | 0.935 |
| **EO%** |  | 1.8 (1.49) | 2.09 (1.96) | 0.108 |
| **BASO%** |  | 0.53 (0.26) | 0.55 (0.29) | 0.298 |
| **ACD (mm)** |  | 3.35 (0.57) | 3.38 (0.59) | 0.653 |
| **LT (mm)** |  | 3.72 (1.56) | 3.71 (1.52) | 0.197 |
| **WTW (mm)** |  | 11.83 (0.45) | 11.75 (0.46) | 0.033 |
| **CCT (μm)** |  | 537.48 (33.4) | 533.89 (34.97) | 0.106 |
| **Operation Time (min)** |  | 61.05 (20.76) | 59.96 (19.38) | 0.818 |
| **Combined Cataract Surgery** | **No** | 143 (89.4%) | 333 (89.3%) | 1.000 |
|  | **Yes** | 17 (10.6%) | 40 (10.7%) |  |
